# Small Molecules Present in the Cerebrospinal Fluid Metabolome Influence Superoxide Dismutase 1 Aggregation

**DOI:** 10.3390/ijms140919128

**Published:** 2013-09-17

**Authors:** Joana S. Cristóvão, Sónia S. Leal, Isabel Cardoso, Cláudio M. Gomes

**Affiliations:** 1 Instituto de Tecnologia Química e Biológica, Universidade Nova de Lisboa, Av. da República, EAN, Oeiras 2784-505, Portugal; E-Mails: jcristovao@itqb.unl.pt (J.S.C.); leal@itqb.unl.pt (S.S.L.); 2 Molecular Neurobiology Unit, Instituto de Biologia Molecular e Celular, Rua do Campo Alegre, 823, Porto 4150-180, Portugal; E-Mail: icardoso@ibmc.up.pt; 3 Escola Superior de Tecnologia da Saúde do Porto, Instituto Politécnico do Porto, Rua Valente Perfeito, 322, Vila Nova de Gaia 4400-330, Portugal

**Keywords:** protein aggregation, amyloid, neurodegeneration, nanoparticle tracking analysis, transmission electron microscopy, dynamic light scattering, small molecules

## Abstract

Superoxide dismutase 1 (SOD1) aggregation is one of the pathological markers of amyotrophic lateral sclerosis (ALS), a fatal neurodegenerative disorder. The underlying molecular grounds of SOD1 pathologic aggregation remains obscure as mutations alone are not exclusively the cause for the formation of protein inclusions. Thus, other components in the cell environment likely play a key role in triggering SOD1 toxic aggregation in ALS. Recently, it was found that ALS patients present a specific altered metabolomic profile in the cerebrospinal fluid (CSF) where SOD1 is also present and potentially interacts with metabolites. Here we have investigated how some of these small molecules affect apoSOD1 structure and aggregation propensity. Our results show that as co-solvents, the tested small molecules do not affect apoSOD1 thermal stability but do influence its tertiary interactions and dynamics, as evidenced by combined biophysical analysis and proteolytic susceptibility. Moreover, these compounds influence apoSOD1 aggregation, decreasing nucleation time and promoting the formation of larger and less soluble aggregates, and in some cases polymeric assemblies apparently composed by spherical species resembling the soluble native protein. We conclude that some components of the ALS metabolome that shape the chemical environment in the CSF may influence apoSOD1 conformers and aggregation.

## 1. Introduction

Superoxide dismutase 1 (SOD1) is a ubiquitous radical scavenger found to aggregate in the motor neurons of patients with both familial and sporadic amyotrophic lateral sclerosis (ALS) [1–7]. This fatal neurodegenerative disease is suggested to be a conformational disorder where proteinaceous aggregates induce specific neuron toxicity and degeneration [8,9] via several proposed mechanisms that include for e.g., organelle dysfunction, proteasome impairment, generation of reactive oxidative species or sequestration of essential cellular components within the aggregates [10]. Although more than 164 mutations in the *sod1* gene have been linked to ALS, it is presently unclear whether mutations are *per se* the requisite for SOD1 pathological aggregation, as not all carriers of *sod1* ALS mutations actually develop the disease [11]. Presently the molecular mechanisms that actually lead to pathological intracellular SOD1 aggregation in ALS remain obscure. Interestingly, recent findings evidenced that extracellular SOD1 aggregates strongly activate microglia [12] thus providing a previously absent link between protein inclusions formation and microglial activation in ALS. Moreover, extracellular SOD1 aggregates penetrate cells and seed aggregation of intracellular soluble protein [13], thus suggesting that extracellular SOD1 aggregates may play a yet unknown role in ALS pathology.

In addition to symptomatic proteinaceous inclusions in motor neurons, ALS patients simultaneously evidence an altered metabolomic profile in the cerebrospinal fluid (CSF) [14–17]. CSF surrounds the central nervous system, rinsing metabolic waste and potentially toxic products away from the brain and spinal cord tissue. Although SOD1 is primarily a cytosolic protein, there is evidence that it is secreted [12,13,18,19], being also detected in the CSF of ALS patients although at levels comparable to those of control individuals [20,21]. However, the effect that small molecules present in the CSF could exert over SOD1 conformation and aggregation propensity remains unaddressed.

The interplay between small molecule metabolites and misfolding-prone proteins affected in folding diseases is potentially very relevant in numerous pathologies, from metabolic disorders to neurodegenerative conditions [22–24]. Small molecules and cofactors can bind to proteins or alter protein hydration patterns, and consequentially influence protein-protein interactions, and modulate the aggregation and structure of aggregates [25,26]. Indeed, there are several published cases in neurodegeneration. For example, in Alzheimer’s Disease (AD) phospholipid metabolites found elevated in AD patients promote Aβ40 aggregation [27] as well as glycine [28], while arginine inhibits Aβ42 aggregation [29]. On the other hand, fructose, sucrose and glucose inhibit or reduce fibrillation of Aβ42 and Aβ40 but promote the formation of oligomers [30]. In ALS, numerous studies are focusing on the use of small molecules as drug-like agents, from molecules capable of decreasing SOD1 expression levels [31,32] to those that would stabilize SOD1 and prevent its aggregation [33].

The cell environment is known to modulate protein stability and folding [34] and it is thus clear that the chemical moiety of the neuronal environment strongly influences the aggregation propensity of SOD1 and this involves small molecules, metal ions and other proteins. For example, calcium is known to be increased in cellular and animal models of ALS [35,36], and recently it has been reported that calcium ions do promote SOD1 aggregation into non fibrillar amyloid, thus suggesting a link to toxic effects of calcium overload in ALS [37]. Since the chemical composition of the CSF in a way reflects that of the central nervous system, it is therefore important to investigate how the ALS metabolomic profile may influence SOD1 aggregation, especially considering that extracellular SOD1 aggregates can be toxic to intracellular soluble SOD1. In this study, we have examined *in vitro* how seven small molecules which are present in the CSF metabolome (lysine, arginine, ornithine, fructose, glyceric acid, erythronic acid and phthalate) influence SOD1 structure and aggregation propensity.

## 2. Results and Discussion

SOD1 is a highly stable homodimeric protein that holds in each monomer a Cu/Zn binuclear site. Mounting evidences suggest that the demetallated SOD1 (apoSOD1) state of the protein is likely the conformer involved in ALS pathological aggregation, as many ALS mutations only present a destabilizing effect on the immature form [38,39] and SOD1 aggregates in transgenic ALS mouse models tend to be metal-deficient [40,41]. Thus, we have carried out this study using the apoSOD1 form of the protein as a model. The selected metabolites that were tested as co-solvents are physiologically present in the human CSF in micromolar concentrations (≈5–200 μM), are essentially hydrophilic and have broad physiological characteristics (Table 1).

We have selected molecules with unaltered (l-ornithine, phthalate, erythronic acid) and decreased levels (l-arginine, l-lysine, glyceric acid, fructose) in sporadic ALS to test their effect on apoSOD1. In addition, we have selected metabolites with different biochemical properties (amino acids, sugar acids, exogenous molecules and monosacharides). This approach, although limited to seven small molecules, allowed us to cover the effects of different small molecules on apo SOD1 aggregation. Interestingly, SOD1 concentration in the CSF has been determined to be ≈10 μM in controls as well as in sporadic and familial ALS cases [20], part of which has been determined to be in a misfolded state [21]. Therefore, we sought to investigate what would be the effect of the CSF metabolites over apoSOD1 in proportions similar to the physiological ones.

### 2.1. Effects on apoSOD1 Structure and Dynamics

**Figure 1:**
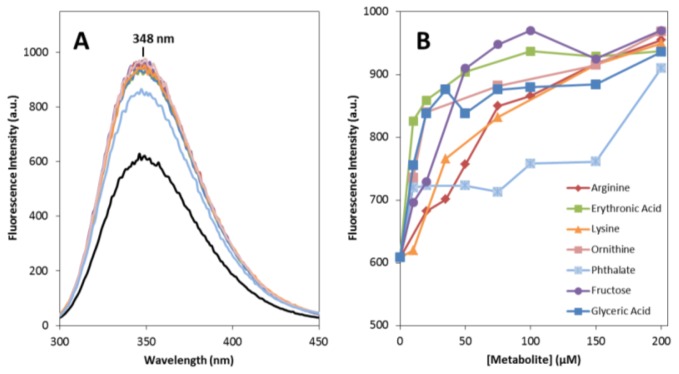
Metabolites impact on apoSOD1 tertiary structure. (**A**) Tryptophan fluorescence emission spectra of 5 μM apoSOD1 (black curve) and upon incubation with 200 μM of each metabolite (colors); (**B**) Concentration dependence of apoSOD1 tryptophan fluorescence emission maximum at 348 nm, for the different metabolites. See experimental section for further details.

To mimic conditions such as those found in the CSF, we have used near physiological concentrations of apoSOD1 (from 5 to 50 μM) and metabolites, which were always present in assays at low micromolar concentrations (up to 200 μM) and never in large molar excess in respect to the protein. Under these conditions, we have observed that the presence of these compounds as co-solvents at metabolite:protein ratios up to 40 has no significant effect on the typical β-sheeted far-UV CD signature of SOD1, suggesting that secondary structure is not perturbed. Some changes were however recorded in the weak broad CD features observed in the 230–260 nm region, suggesting some alterations on the protein tertiary structure (not shown). In order to further investigate this aspect we monitored tryptophan fluorescence of apoSOD1 as a function of different concentrations of metabolites, from 5 to 200 μM (Figure 1).

**Figure 2:**
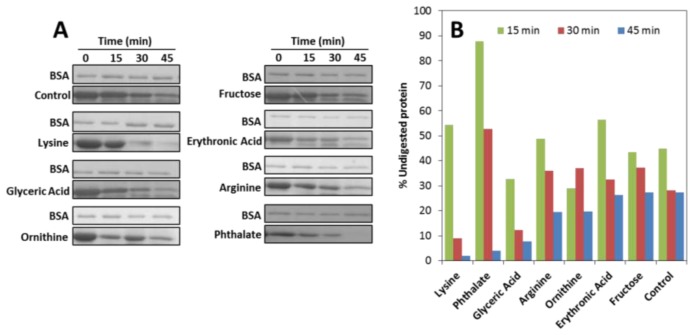
Metabolites increase apoSOD1 proteolytic susceptibility. (**A**) SDS-PAGE analysis of a time course trypsin digestion experiment (50 μM apoSOD1 and 200 μM of metabolites); (**B**) Percentage of digested apoSOD1 in the presence of the different metabolites after 15, 30 and 45 min of incubation with trypsin. See experimental section for further details.

SOD1 has only a single tryptophan residue (Trp-32) which is located in the outer surface of the eight stranded antiparallel β-barrel and is thus unusually solvent exposed. In the presence of any of the seven metabolites no shift in the emission maximum (λ_max_ 348 nm) is observed but a significant increase in fluorescence intensity is noted. This effect is concentration dependent in the 5 to 200 μM range and it seems to stabilize at metabolite to protein ratios above 20. This result suggests an effect of the small molecule co-solvents in apoSOD1 tertiary structure, probably through the establishment of conspicuously weak unspecific interactions between the compounds and the protein, like those observed in osmolyte effects [43]. This weak interaction is corroborated by the fact that metabolites in conditions identical to those in which Trp emission changes have no effect on the thermal stability of SOD1 at concentrations up to 200 μM, as determined from differential scanning fluorimetry (DSF) (not shown). To further explore how metabolites influence SOD1 tertiary structure and conformational dynamics we have monitored the proteolytic susceptibility towards trypsin, as a means to investigate if the presence of the different metabolites influences the protein breathing dynamics (Figure 2). The results show that most metabolites increase SOD1 susceptibility to proteolysis, likely by inducing tertiary changes that increase the protein dynamics and facilitate the access of trypsin to otherwise inaccessible cleavage sites, thus enhancing the degradation rate. Interestingly, most of the Lys (Lys23, Lys30, Lys36, Lys121, Lys127, Lys136) and Arg residues (Arg143) that are cleavable by the protease, as well as the Trp32 are localized within SOD1 β-barrels that exhibit partial unfolding at physiological temperature (37 °C) in apoSOD1 [44]. This suggests that metabolites impact significantly in this highly dynamic region of apoSOD1.

Finally, we have examined if the effects on tertiary structure and protein dynamics would result in an increased exposure of hydrophobic regions. To verify if the metabolites influence apoSOD1 hydrophobicity we have used 1-anilino-8-naphthalene sulfonate (ANS) as a probe. Upon binding to hydrophobic regions ANS becomes fluorescent and is thus a useful reporter to detect exposed hydrophobic patches in structured proteins, including β-sheeted structures [45]. It is known that apoSOD1 binds ANS and has an aberrant hydrophobicity that could potentiate self-interactions and aggregation [46,47]. However, the presence of metabolites did not result in an enhancement of ANS fluorescence (not shown), indicating that these compounds do not act by exposing hydrophobic segments. Taken together, the results presented in this section suggest that metabolites at low concentrations likely promote a perturbation of apoSOD1 tertiary structure, via unspecific interactions which result in apoSOD1 increased flexibility. This effect may thus broaden apoSOD1 native-like dynamic conformations that may influence the aggregation process.

### 2.2. Effects on apoSOD1 Aggregation Kinetics

**Figure 3:**
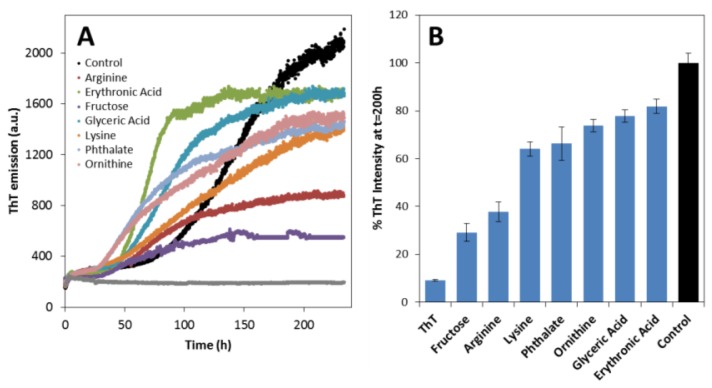
Metabolites promote aggregation of apoSOD1. (**A**) Aggregation kinetics of 50 μM apoSOD1 monitored by ThT fluorescence emission, in the absence (black) and in presence (colors) of 200 μM metabolites; (**B**) Plot of ThT fluorescence intensity at 200 h (from traces in panel **A**). Both panels are representative of 3 independent measurements with 50 μM apoSOD1 at pH 7.4 under agitation at 37 °C with and without 200 μM of each metabolite. The ThT bar represents the fluorescence intensity of the probe in the absence of protein and metabolites. See experimental section for further details.

In the next stage, we focused on investigating if metabolites influence SOD1 aggregation. The aggregation kinetics of apoSOD1 was followed by monitoring Thioflavin-T (ThT) binding, a fluorescent probe that recognizes diverse amyloid like fibrils and amorphous aggregates [48–50], as well as oligomers and protofibers [51,52]. We have used an established experimental condition (50 μM protein in 50 mM Tris at pH 7.4 with agitation at 600 rpm at 37 °C) in which β-aggregates and fibrils are formed from apoSOD1 within about 8 days [37], and the effect of metabolites was tested at a metabolite:protein ratio of 4 (Figure 3).

Under these conditions the aggregation of apoSOD1 can be mechanistically described as a nucleation-dependent aggregation process characterized by a sigmoidal type transition from which the lag phase time (*t*_lag_) and aggregation rate can be determined (Table 2).

Metabolites substantially decreased the lag phase of apoSOD1 aggregation, suggesting that these molecules promote unfavorable associations of apoSOD1 to form an organized oligomerization nucleus (Figure 3A). Fructose, ornithine and phthalate had the most impressive effect decreasing the lag time in 71, 68 and 61 h respectively. However, these compounds had a more discrete impact on apoSOD1 aggregation rate: erythronic acid, ornithine, phthalate and arginine resulted in the highest aggregation rates (from 0.054 to 0.093 h^−1^) followed by glyceric acid and fructose (0.044 h^−1^), which was only slightly higher than that of the control (0.040 h^−1^). Only lysine resulted in a slower aggregation process from which a lag phase could not be accurately determined. Curiously, the intensity of ThT binding at the stationary phase of apoSOD1 aggregation kinetics (≈200 h), is consistently depressed by the presence of the metabolites, ranging from a 20% decrease with glyceric and erythronic acid to 70% with fructose (Figure 3B). ThT intensities are influenced by the morphology of fibrils and β-aggregates [53] in which ordered intermolecular β-sheet structures are suggested to bind preferentially ThT [45]; thus these small molecules either modulate apoSOD1 aggregation into less ordered β-sheet aggregates or/and lead to the formation of a reduced amount of total aggregates.

Determined from fits to Equation 1. See experimental section for details. n.d., not determined. Since the analyzed metabolites have distinct molecular structures and chemical properties, it was somehow unexpected that all co-solvents boost the onset of apoSOD1 aggregation by decreasing the lag phase. Arginine, lysine and ornithine as amino acids and fructose as a sugar (Table 1), are generally classified as stabilizing osmolytes, thought to stabilize globular proteins by favoring compaction through preferential exclusion [25]. This is unlikely the case with apoSOD1, as at the tested concentrations these compounds did not increase protein thermal stability but rather promoted intermolecular aggregation. The molecular basis underlying the effect of these positively charged amino acids may derive from electrostatic interactions established with the protein chains, as SOD1 has a negative net charge at physiological pH. Aggregation would thus be favored through neutralization of native apoSOD1 repulsive charges that could decrease solubility and/or promote aberrant protein-protein interactions. Indeed, many of the ALS pathological mutations are suggested to lead to increased aggregation propensity due to these effects [54]. In addition, arginine which is frequently considered as a beneficial aggregation suppressor by enhancing the solubility of proteins [55,56], actually promoted apoSOD1 aggregation, an effect also evidenced in other proteins such as bovine serum albumin and β-lactoglobulin [57]. The versatility of arginine to suppress/promote protein aggregation is thought to rely on the preferential binding of its methyl guanidinium group, which may either interact with aromatic side chains (suppressing aggregation) or with acidic side chains (promoting aggregation). SOD1, similarly to bovine serum albumin and β-lactoglobulin is an acidic protein, thus suggesting that the enhancement in SOD1 aggregation may be related with the bidentate capacity of the arginine guanidinium group to interact simultaneously with two acidic residues, acting as a bridge and promoting protein-protein interactions. In agreement, guanidine hydrochloride that also presents a guanidinum group was evidenced to promote apoSOD1 aggregation [58,59].

### 2.3. Effects on the Size, Solubility, and Morphology of apoSOD1 Aggregates

Here we have thoroughly evaluated the impact of each metabolite on the size, solubility and morphology of the apoSOD1 aggregates formed at the stationary phase of the aggregation kinetics, using a combination of biophysical, biochemical and imaging techniques.

**Figure 4:**
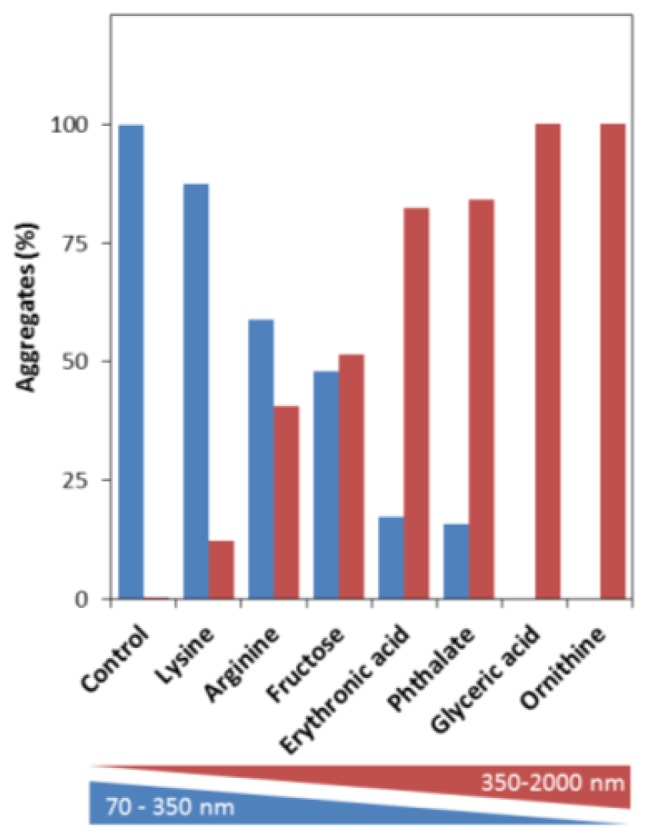
Metabolites boost the formation of larger apoSOD1 aggregates. Nanoparticle Tracking Analysis (NTA) of the distribution of total apoSOD1 aggregates generated from 50 μM apoSOD1, alone and in the presence of 200 μM of each metabolite after incubation at pH 7.4 under agitation at 37 °C for 200 h (metabolite:protein = 4). Blue bars correspond to the distribution of small aggregates (70–350 nm) and red bars correspond to larger aggregates (350–2000 nm). See experimental section for further details.

To analyze the total distribution profile of protein aggregates in solution we have used nanoparticle tracking analysis (NTA), an innovative technique that accurately measures the size and distribution of particles in very heterogeneous formulations, such as aggregated protein solutions [60]. Briefly, this method measures the Brownian motion of nanoparticles which are analyzed by video allowing individual particle positional changes to be evaluated in two dimensions, from which the diffusion constant can be measured, and from there the hydrodynamic diameter of the particle can be then determined [60]. A comparative NTA analysis of apoSOD1 aggregates formed in the presence and absence of metabolites shows that these substantially influence the distribution of apoSOD1 aggregates (Figure 4). Whereas in the absence of metabolites (control), apoSOD1 mostly develops aggregates with hydrodynamic radii lower than 350 nm, in the presence of the tested organic compounds apoSOD1 evidenced a consistent shift towards the formation of larger aggregates (350–2000 nm) (see supplementary materials for representative NTA videos). In particular, the most extreme cases were those in which glyceric acid and ornithine were present which resulted essentially in 100% formation of particles larger than 350 nm. Erythronic acid and phthalate promoted the formation of mostly larger aggregates (>80%), whereas with fructose and arginine there was nearly an even distribution. The condition closer to that of control apoSOD1 was observed when lysine was present, as in this case only ≈10% of the larger species were observed.

**Figure 5:**
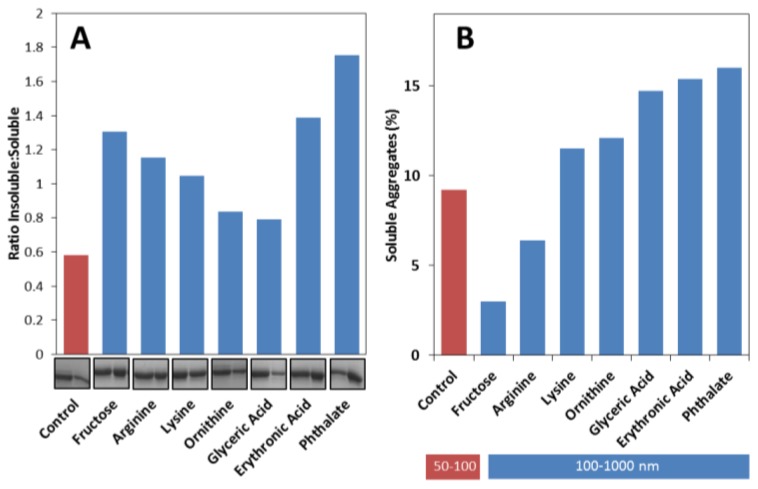
Metabolites influence apoSOD1 aggregation towards insoluble aggregates and increase the size of soluble aggregates. (**A**) Ratio of soluble and insoluble aggregates formed from 50 μM apoSOD1 with 200 μM of each metabolite. SDS-PAGE gel: first band correspond to soluble aggregates and second band corresponds to insoluble aggregates, after incubation of apoSOD1 under agitation at 37 °C for 200 h; (**B**) DLS size distribution of soluble aggregates of apoSOD1 alone and with 200 μM of each metabolite after incubation at pH 7.4 under agitation at 37 °C for 200 h. Red bar correspond to apoSOD1 particles (50–100 nm) and blue bars correspond to larger aggregates formed in the presence of metabolites (100–1000 nm). See experimental section for further details.

Considering these results we have then decided to analyze if apoSOD1 aggregates formed in the presence of metabolites are mostly soluble or insoluble. For the purpose, samples collected at the plateau phase (≈200 h) were centrifuged to resolve insoluble and soluble fractions, which were then analyzed by SDS-PAGE and quantitated in order to determine the insoluble:soluble ratio (Figure 5A). The results obtained showed that metabolites influence apoSOD1 aggregation pathway towards the formation of insoluble aggregates. In the absence of metabolites, most of the aggregates formed are soluble, as indicated by an insoluble:soluble ratio below 1 (≈0.6). However, the presence of metabolites increases this ratio in the following order: glyceric acid ≈ ornithine < lysine < arginine < fructose < erythronic acid < phthalate. From these only ornithine and glyceric acid have ratios below 1, which means that most of the molecules shift the aggregation pathway towards insoluble aggregates rather than soluble ones.

We then analyzed the pool of soluble aggregates that are formed at the stationary phase, focusing on the smaller particles and to investigate if any SOD1 dimer persists in solution. From a NTA analyses it is not possible to evaluate the percentage of SOD1 dimer (≈5.5 nm) that remains in solution and therefore estimate the impact of the metabolites in the total percentage of SOD1 aggregation, as NTA is only sensitive and accurate to track particles larger than 30 nm [60]. For the purpose, we have used DLS, which measures fluctuations in scattered light intensity due to diffusing particles; sample centrifugation and filtering to remove large interfering scatterers from suspension allowed focusing on the soluble particles

DLS analysis indicates that dimeric apoSOD1 is the most predominant state in solution, irrespective of the presence of metabolites, yielding 85%–95% of total protein. Therefore, metabolites marginally impact on the total percentage of soluble aggregates, causing small variations in respect to the control: fructose and arginine decrease the total percentage of soluble aggregates (less than 4%) while all other metabolites slightly increase the fraction of soluble aggregates (up to 8%) (Figure 5B). However, the distribution of soluble aggregates is distinct if metabolites are present or absent: whereas in the absence of added metabolites (control) most of the soluble aggregates detected are within the 50–100 nm interval, in their presence the predominant distribution is observed for particles ranging from 100 to 1000 nm in hydrodynamic radius (Figure 5B). Curiously, fructose and arginine are the metabolites that result in lower amounts of DLS detected soluble aggregates, while yielding insoluble:soluble ratios higher than 1. These combined results indicate that these compounds have a stronger influence on apoSOD1 aggregation towards insoluble aggregates

**Figure 6:**
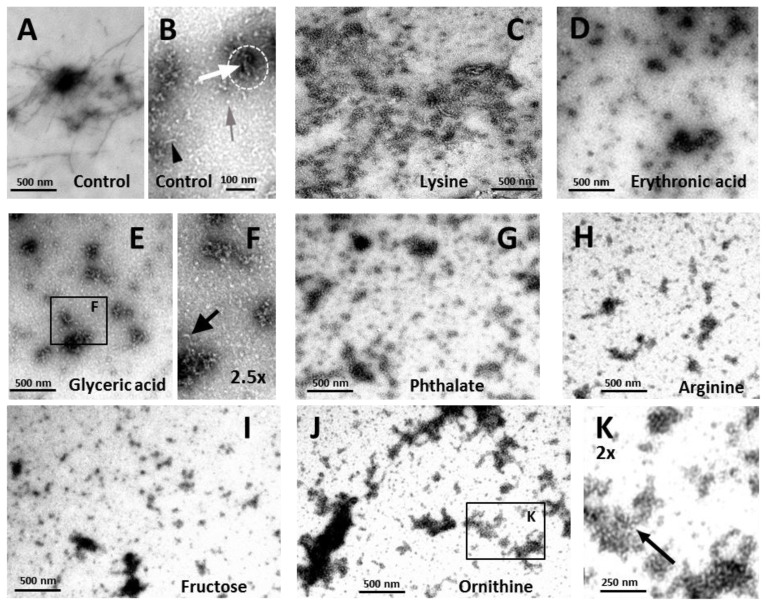
Metabolites modulate the morphology of apoSOD1 aggregates. TEM images of apoSOD1 with or without metabolites at a metabolite:protein ratio of 4, after incubation at pH 7.4 under agitation at 37 °C for 200 h. (**A**,**B**) Control, apoSOD1 with no added metabolite; (**C**) +lysine; (**D**) +erythronic acid; (**E**) +glyceric acid; (**F**) +glyceric acid, magnification of panel E; (**G**) +phthalate; (**H**) +arginine; (**I**) +fructose; (**J**) +ornithine; (**K**) +ornithine, magnification of panel J. See experimental section for further details.

To further characterize the species formed during the incubation of apoSOD1 with the metabolites, transmission electron microscopy (TEM) analysis was performed on the samples after the stationary phase was reached (Figure 6).

Under these conditions, only discrete structural differences were observed between samples. Control apoSOD1 alone appeared as fibrils (Figure 6A) and aggregates (Figure 6B, white arrow) in characteristically pre-fibrillar assemblies (Figure 6B, doted white circle); short fibrils approximately 8 nm wide were easily detected (Figure 6B, black arrowhead), as well as oligomers of similar diameter (Figure 6B, grey arrow). The presence of lysine did not produce major distinct species although the preparations seemed more delayed than that of the control apoSOD1 alone, as only large aggregates were visualized. These aggregates seemed to be less structured than the ones from the control and no further species, namely short fibrils or oligomers, were distinguishable (Figure 6C). In contrast, abundant very short fibrils and aggregate assemblies were observed in the presence of erythronic acid (Figure 6D). The presence of glyceric acid (Figure 6E,F) and phthalate (Figure 6G) resulted in the prominent formation of aggregates and oligomers, although short fibrils were also identified (Figure 6F, black arrow). On the other hand, in the presence of arginine (Figure 6H), fructose (Figure 6I), erythronic acid (Figure 6D) and ornithine (Figure 6J,K), although apoSOD1 was mostly oligomeric very short fibrils were visualized more abundantly, indicating that the aggregation process was slightly accelerated in the presence of these metabolites. This is indeed in agreement with the ThT binding kinetics, as higher aggregation rates and decreased lag times were observed when these metabolites were present. Noteworthy, in some of the preparations, namely in the presence of arginine, fructose and ornithine, polymeric formations were observed, as illustrated for ornithine. The basic unit of these formations appears as a spherical species that resembles soluble native protein (Figure 6K, black arrow). Interestingly, the filamentous assembly of SOD1 has been previously proposed on the basis of crystal packing observed during structure determination of different ALS-related SOD1 variants namely “linear amyloid-like” and “zigzag” filaments which could self-assembly into higher order oligomers [61]. These filaments would result from extensive interactions between loop and β-barrel elements of neighboring SOD1 molecules, as a result from gain of interaction between SOD1 dimers would leads to higher-order arrays [61]. To the best of our knowledge, such structures have not been reported by ultrastructural imagining methods, even though SOD1 fibers have been thoroughly described [37,58,62]. We can speculate that the polymers observed in the presence of some of the metabolites could result from metabolite-induced conformational changes in apoSOD1 that would generate similar “gain-of-interaction” interfaces that would promote assembly into polymeric filaments composed by soluble native protein.

## 3. Experimental Section

### 3.1. Chemicals and Proteins

All reagents were of the highest commercially available grade. SOD1 expression and purification were performed as in [63] and preparation of apoSOD1 was made as in [64]. All buffers were passed through Chelex resin (Bio-Rad, Hercules, CA, USA) to remove contaminant trace metals. Quantification of metals in apoSOD1 was confirmed spectrophotometrically using the Zincon method [65]. Concentration of SOD1 was determined using the extinction coefficient 10,800 cm^−1^M^−1^ at 280 nm.

### 3.2. Fluorescence Spectroscopy

Fluorescence spectroscopy was performed using a Cary Eclipse instrument (Agilent, Santa Clara, CA, USA) equipped with a Peltier temperature control. For tryptophan emission, excitation wavelength was set at 280 nm; slits were set to 10 nm for excitation and emission. Experiments were performed with 5 μM apoSOD1 in 50 mM Tris pH 7.4 and 200 μM of each metabolite, after overnight incubation (~14 h).

### 3.3. Circular Dichroism (CD)

Far UV CD analysis was performed using a Jasco J-815 spectropolarimeter (Jasco UK, Essex, UK) equipped with a Peltier-controlled thermostated cell support. Spectra were recorded with 6 μM apoSOD1 in 50 mM Tris pH 7.4, previously incubated overnight with 200 μM of each metabolite.

### 3.4. Differential Scanning Fluorimetry

Differential scanning fluorimetry (DSF) was used to determine the melting temperatures (*T*_m_) of apoSOD1 in the presence of metabolites. Prior to measurements 6 μM of apoSOD1 was incubated overnight with 200 μM of each metabolite at room temperature. No precipitation was observed. The samples were distributed into PCR plates (Bio-Rad, Hercules, CA, USA) and mixed with Sypro Orange 5× (Invitrogen, Carlsbad, CA, USA). The plates were sealed with optical quality sealing tape (Bio-Rad, Hercules, CA, USA) and measurements were made in an iCycler iQ Real-Time PCR instrument (Bio-Rad, Hercules, CA, USA) setting the excitation filter from 530 to 560 nm and the emission filter from 575 to 595 nm. The used temperature range was 20 °C to 90 °C, with a linear increment at 1 °C·min^−1^.

### 3.5. Trypsin Limited Proteolysis

Digestion of apoSOD1 in the presence of metabolites was performed by incubation of the samples with trypsin (bovine pancreas trypsin; PVL) at 37 °C in 50 mM Tris pH 7.5 at a SOD1:trypsin ratio = 50. As a control, identical samples without addition of trypsin were also submitted to the same procedure. Aliquots with 50 μM of apoSOD1, 200 μM of different metabolites and 1 μM trypsin were sampled at different time points (0; 15; 30 and 45 min). The reaction was stopped by the addition of SDS-PAGE loading buffer (2% SDS and 5% β-mercaptoethanol). Two point five micromolar bovine serum albumin (BSA) was included in gels as internal loading control. The products of the proteolysis reaction were analyzed by 12% SDS/PAGE, which were stained with Coomassie Brilliant Blue R-250.

### 3.6. SOD1 Aggregation Assays

Real time ThT fluorescence (480 nm) was recorded using a BMG Fluostar Optima fluorescence plate reader upon excitation at 440 nm. All the assays were performed in black 96 well plates (Nunc, #732-2701, Denmark) and subjected to agitation at 600 rpm prior to each fluorescence measurement. Experiments were carried out with 50 μM of apoSOD1 and 100 μM ThT in 50 mM Tris pH 7.5 at 37 °C. The concentration of metabolites used was 200 μM. Samples were prepared as triplicates with 200 μL as final sample volume in each well, which also included one teflon bead (1/8 inch in diameter). Analysis of the aggregation curves was performed by fitting the data to the following equation:

(1) $$Y=(y_{i}+m_{i}x)+\frac{\left(v_{f}+m_{i}x\right)}{\left(1+\exp^{-(X-\frac{X_{o}}{\tau })}\right)}$$

where *Y* corresponds to fluorescence intensity, *x* is the time and *X*_0_ corresponds to the time of half-height of fluorescence intensity (*t*_50_). The *lag phase* is calculated by *t*_lag_ = *X*_0_ − 2τ and the apparent rate by *K_app_* = 1/τ.

### 3.7. Dynamic Light Scattering (DLS)

DLS measurements were carried out in a Malvern Zetasizer Nano ZS instrument equipped with a 4-megawatt He-Ne laser (632 nm). Fifty micromolar apoSOD1 with 200 μM of each metabolite in 50 mM Tris at pH 7.4 were incubated at 37 °C with agitation set to 600 rpm. After 200 h of incubation, samples were centrifuged at 20,000 × *g* for 30 min to obtain only the soluble aggregates. The samples were measured in a 45 μL quartz cuvette (Hellma, Müllheim, Germany). The operating procedure was set to 3 runs, each being averaged for 11 measurements. The resulting data were analyzed using the DTS software v6.32 (Malvern, Worcestershire, UK, 2012).

### 3.8. Nanoparticle Tracking Analysis (NTA)

NTA measurements were carried out in a Nanosight NS500 equipped with a 405 nm laser. Fifty micromolar apo SOD1 with 200 μM of each metabolite in 50 mM Tris at pH 7.4 were initially filtered through a 0.45 μM filter and incubated at 37 °C with agitation set to 600 rpm. After 200 h of incubation, the samples were diluted (1:5) and measured. All measurements were collected with a camera level set to 15 to allow for the detection of smaller particles. Data were captured and analyzed using NTA software v. 2.3 (Nanosight, Limited, Amesbury, UK, 2013).

### 3.9. Quantification of Insoluble Aggregates

The levels of soluble and insoluble aggregates were analyzed using 12% SDS-PAGE. To separate the insoluble fraction from the total fraction a centrifugation at 20,000× *g* was carried out during 30 min. After centrifugation, the supernatants (soluble aggregates) were transferred to a fresh tube and pellets (insoluble fraction) were resuspended in loading buffer. The absolute intensity of each band was quantified using Image Lab (Biorad, Hercules, Canada) subtracting the mean intensity from BSA bands.

### 3.10. Transmission Electron Microscopy

For visualization by TEM, 5 μL of 50 μM apoSOD1 aggregates formed with and without 200 μM of each metabolite were absorbed to carbon-coated collodion film supported on 400-mesh copper grids, and negatively stained with 1% uranyl acetate. The grids were exhaustively visualized with a Jeol microscope (JEM-1400, Tokyo, Japan), operated at 80 kV.

## 4. Conclusions

In this study, we have investigated the effects of components of the CSF metabolome over SOD1, a protein that undergoes toxic aggregation in ALS, a fatal neurodegenerative disease. Recent studies have indicated specific level variations of some CSF metabolites in association with ALS pathology, and we have designed an initial study to investigate to which extent the chemical composition of the CSF influence apoSOD1 aggregation. What we conclude is that apoSOD1 aggregation is enhanced and modulated in the presence of all analyzed metabolites (lysine, arginine, ornithine, fructose, glyceric acid, erythronic acid and phthalate), irrespective of their levels in ALS. Therefore, a correlation between deregulation of CSF metabolites and SOD1 aggregation is very unlike to be established for ALS. Nevertheless, this study unveils the possibility that small molecules at physiological concentrations may play a role as modulators of SOD1 aggregation.

## Figures and Tables

**Table 1:** Metabolites present in the cerebrospinal fluid (CSF) metabolome.

Compound	Compound properties		CSF levels (μM)		Sporadic ALS

	Type	Physiological charge	Normal	Altered	
l-Arginine		+1	15–25	6–21	decreased
l-Lysine	amino acid	+1	18–30	45	decreased
l-Ornithine		+1	4–8	7–8	unchanged

Glyceric acid	sugar acid	−1	34	n.a.	decreased
Phthalate	exogenous	−2	n.a.	n.a	unchanged

Fructose	monosaccharide	0	160–240	n.a.	decreased
Erythronic Acid		−1	5	n.a	unchanged

Normal and altered metabolite concentrations (approximated to the closest integer values) and predicted physical properties were extracted from the Human Metabolome Database Version 3.5 at http://www.hmdb.ca/ [42]. Information on relative variation of metabolites in sporadic ALS is taken from [14]. n.a., not available.

**Table 2:** Kinetic data from ThT SOD1 aggregation curves.

Compound	Lag time *t*_lag_, h	Aggregation rate *k*_app_, h^−1^
Control (no compound)	88	0.040
Erythronic Acid	47	0.093
l-Ornithine	20	0.062
Phthalate	24	0.059
l-Arginine	41	0.054
Glyceric acid	39	0.044
Fructose	17	0.044
l-Lysine	n.d.	0.019

## Supplementary Files

Supplementary File 1 Supplementary (ZIP, 27831 KB)
